# 

**Published:** 1923-02

**Authors:** 


### HOSPITAL and HEALTH REVIEW

Established 1886 No. 1859. Vol. LXXII.

New Series. Vol. II. No. 17 February 1923 Price Sixpence

CONTENTS.

| | PAGE |
|---|---|
| Health and the Roof Tree | 95 |
| Notes and Comments—The Hospital Saving Association—Therms and Explosions—The Jenner Centenary—The Doncaster Scheme—The Overlapping of Public Assistance—Cadet Corps as Health Centres—A Research Clearing-House—The Unceasing Annuitant—Lowered German Vitality—Public Health in Czechoslovakia—Wasted Appeals—Medical Inspection of Teachers—Country Houses as Institutions | 97 |
| The Combined Appeal: Has it Been Successful ? | |
| —Yes By the Hon. Sir Arthur Stanley | 101 |
| —No By E. W. Morris | 102 |
| A Clean Milk Opportunity | 103 |
| The Organisation of Panel Practice By R. W. Harris | 104 |
| The Public Health—Child Welfare in the West Riding | 105 |
| The Future of Voluntary Hospitals | 107 |
| National Health Insurance Notes | 109 |
| Regional Planning for Health and Beauty—A Vision of a New South Yorkshire | 110 |
| Labour's Health Policy | 111 |
| Hospital Finance | 112 |
| Boys and Girls in Secondary Schools | 113 |
| Correspondence | 114 |
| Reviews of Books | 117 |
| Hospital and Health News | 121 |
| Government Publications | 122 |
| Echoes of the Month | 123 |
| Public Health in Parliament | 125 |
| Recent Appointments | 126 |

